# Comparative Analysis of Antifungal Susceptibility in Trichophyton Species Using Broth Microdilution Assay: A Cross-Sectional Study

**DOI:** 10.7759/cureus.77113

**Published:** 2025-01-07

**Authors:** Anshu Singh, Amber Prasad, Gagandeep Singh, Neirita Hazarika, Pratima Gupta, Neelam Kaistha

**Affiliations:** 1 Microbiology, All India Institute of Medical Sciences, Rishikesh, Rishikesh, IND; 2 Microbiology, All India Institute of Medical Sciences, New Delhi, New Delhi, IND; 3 Dermatology, Venereology, and Leprosy, All India Institute of Medical Sciences, Guwahati, Guwahati, IND; 4 Microbiology, All India Institute of Medical Sciences, Deoghar, Deoghar, IND

**Keywords:** amphotericin and terbinafine, antifungal susceptibility, culture outcomes, dermatophyte, koh positivity, mics

## Abstract

Background

Dermatophyte infections, predominantly caused by *Trichophyton* species, are a global health concern, particularly in tropical regions. The rising prevalence of resistant strains, due to the misuse of antifungal agents, necessitates robust antifungal susceptibility testing. This study aimed to evaluate antifungal susceptibility patterns of clinical isolates using the broth microdilution (BMD) assay to guide treatment strategies effectively.

Methods

A cross-sectional study was conducted at the All India Institute of Medical Sciences (AIIMS), Rishikesh, for over two years, including 184 clinical samples from patients with recalcitrant dermatophytosis. Samples were collected aseptically, and antifungal susceptibility testing was performed using the BMD assay. Minimum inhibitory concentration (MIC) values were analyzed for five drugs: amphotericin, terbinafine, griseofulvin, itraconazole, and fluconazole. Associations with patient demographics, potassium hydroxide (KOH) positivity, and culture outcomes were statistically assessed.

Results

Amphotericin showed the lowest mean MIC (0.57 µg/mL), while fluconazole exhibited the highest (7.70 µg/mL). Amphotericin and terbinafine achieved 100% inhibition, while other drugs showed 80% inhibition. Gender had no significant effect on MIC values. KOH positivity and culture outcomes significantly influenced MICs, with species-specific patterns observed. For instance, *T. mentagrophytes* and *T. indotineae *were linked to higher MICs for fluconazole and griseofulvin.

Conclusion

The study underscores the need for species-specific antifungal susceptibility testing to monitor resistance trends. Results focused on unexpected resistance to typically effective drugs like terbinafine or fluconazole. Routine testing and targeted stewardship are essential to improve therapeutic outcomes for dermatophytosis.

## Introduction

Dermatophyte infections, predominantly caused by *Trichophyton* species, are a growing global health concern, especially in tropical and subtropical regions where warm and humid conditions favor fungal growth and transmission. These infections, commonly presenting as tinea, affect keratinized tissues such as the skin, hair, and nails, and can result in chronic, recurrent conditions and significant morbidity if left untreated or managed ineffectively [1]. The increasing prevalence of these infections, compounded by the widespread misuse and overuse of antifungal agents, has contributed to the emergence of resistant *Trichophyton* strains, posing challenges for effective clinical management [2]. The declining efficacy of frontline antifungal agents, such as azoles and allylamines, has been documented globally, with resistance rates varying across regions and species [3]. These trends emphasize the challenges posed by the rise in antifungal resistance due to overprescription or misuse of treatments and the urgent need for robust antifungal susceptibility testing to inform appropriate treatment strategies. The broth microdilution (BMD) assay, endorsed by the Clinical and Laboratory Standards Institute (CLSI), is a gold standard method for determining minimum inhibitory concentrations (MICs) of antifungal drugs [4]. Its quantitative and reproducible results are crucial for identifying emerging resistance patterns and guiding therapeutic interventions. Recent studies have highlighted significant variability in antifungal susceptibility among *Trichophyton* species, with notable resistance to terbinafine, a commonly prescribed antifungal, in certain geographic regions [5,6]. These findings underscore the importance of species-specific monitoring and susceptibility profiling. This cross-sectional observational study aims to conduct a comparative analysis of antifungal susceptibility in clinical isolates of *Trichophyton* species using the BMD assay. The study's findings will contribute to a deeper understanding of resistance patterns and support evidence-based antifungal stewardship to combat dermatophytosis effectively. The primary objective of this study is to evaluate the antifungal susceptibility and resistance profiles of *Trichophyton* species isolated from dermatophyte infections in order to inform more targeted treatment strategies. 

## Materials and methods

It was a cross-sectional study performed at the All India Institute of Medical Sciences (AIIMS), Rishikesh, over two years. This study started from 22 August 2022 to 7 September 2024.

Sample size

The study is based on a population size (N) of 660, which is derived from assuming 330 samples per year over a study duration of two years. The hypothesized percentage frequency of the outcome factor in the population (p) is 83%, with an absolute precision of 5%. The design effect (DEFF) for cluster surveys is set at 1. For a 95% confidence level, the required sample size is 164. Assuming 10% sample wastage, estimated sample size was 183 [7].

Inclusion criteria

All samples (nail, skin, hair) of clinical cases of recalcitrant dermatophytosis were obtained from patients who attended the dermatology department of AIIMS Rishikesh.

Exclusion criteria 

Patients with naive infections.

Sample collection

Aseptic conditions were used to collect the samples. Before gathering a clinical specimen, preferably from the expanding edge of lesions, the affected location was cleaned with alcohol.

Skin scrapings were gathered from center to edge, crossing boundaries with the use of a sterile scalpel blade held at an angle of 90⁰ to the skin surface. When hair involvement occurred, we plucked the affected hair instead of clipping it. Depending on the type of nail infection, nail clippings were taken from the relevant location.

The material was gathered into folded squares of black paper, which allowed the specimen to dry, decreased bacterial contamination, and created conditions that allowed the specimens to be kept for extended periods of time without significantly compromising the fungal agents' vitality.

Direct microscopy

Potassium hydroxide (KOH) wet mount: The cement substance that keeps keratinized cells together dissolves, and protein waste is softened and digested by the aqueous potassium hydroxide (KOH). On a microscopic slide, the specimen is submerged in a 10% KOH drop and covered with a cover slip. In clinical specimens, the fungal components become easily visible and rather transparent.

Depending on the type of clinical material, the KOH concentration can be raised; for solid specimens, it can reach 40%, while for soft specimens, it can reach 10%.

Culture

Regardless of the results of a direct examination, the clinical specimens ought to be inoculated on fungal culture media. On Sabouraud dextrose agar (SDA) supplemented with antibiotics and cycloheximide, dermatophytes can grow readily. The media are incubated at three different temperatures: 25°C, 30°C, and 37°C.

It often takes between ten days and three weeks for the growth to occur. Colony morphology, lactophenol cotton blue (LPCB) mount, and slide culture were performed to examine the morphology of micro- and macroconidia following growth.

Antifungal susceptibility testing

The Clinical and Laboratory Standards Institute (CLSI) criteria, document M38-A2, for filamentous fungi, were followed in the performance of the in vitro antifungal susceptibility assay. The antifungal agent stock solutions were made for the medications itraconazole (Himedia), fluconazole (Himedia), griseofulvin (Sigma Aldrich), terbinafine (Sigma Aldrich), and amphotericin-B (Himedia). Antifungal susceptibility testing was conducted using Rose Parker Memorial Institute-1640 (RPMI-1640) as a growth medium (pH 7.0 ± 0.1).

Weighing Antifungal Powders

Assay all antifungal agents for standard units of activity. The assay units can differ widely from the actual weight of the powder and often differ within a drug production lot. Thus, a laboratory must standardize its antifungal solutions based on assays of the lots of antifungal powders used.

Use either of the following formulas to determine the amount of powder or diluent needed for a standard solution:

Weight (mg) = Volume (mL) × Concentration (µg/mL) ÷ Potency (µg/mg)

or

Volume (mL) = Weight (mg) × Potency (µg/mg) ÷ Concentration (µg/mL)

The antifungal powder should be weighed on an analytical balance that has been calibrated by approved reference weights from a national metrology organization. Usually, it is advisable to accurately weigh a portion of the antifungal agent in excess of that required and to calculate the volume of diluent needed to obtain the concentration desired.

Dermatophytic strains were cultivated on SDA for seven to 14 days to create stock inocula. Colonies were then submerged in 5 ml of sterile water, and the suspension was created by gently probing the surface with the tip of a sterile device. After transforming the suspended mixture in a sterile tube and letting it settle for fifteen minutes, the upper homogenous suspension was employed for additional testing.

The inoculum concentration was adjusted using a spectrophotometer (530 nm) with an optical density of 0.09-0.11 to get the final concentration of 1×103 to 3×103 CFU/mL. A sterile 96-well microtiter plate with a flat bottom was used to inoculate the prepared solution. Around 100 µl of the conidial suspension in RPMI 1640 was applied to each well as an inoculant, and 100 µl of diluted medication was administered to each well in turn. Additionally, sterility and growth control wells were installed. For four days, every microtitre plate was incubated at 37°C. The control was *Trichophyton* mentagrophytes ATCC MYA-4439 [8].

Analytical statistics

The mean, standard deviation, and standard error were used to measure each quantitative variable. For categorical variables, frequencies and proportions were explained. At the two-sided significance level of p < 0.05, all statistical tests that were used were evaluated. To investigate the relationship between the variables, chi-square tests were used.

## Results

**Figure 1:**
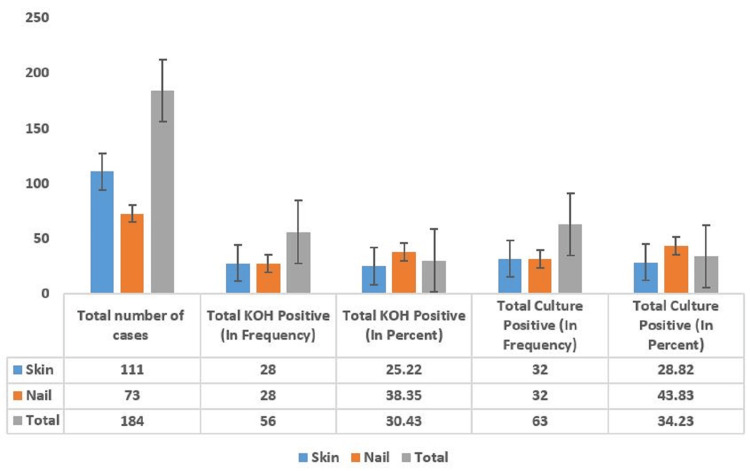
Total number of clinical samples according to KOH and culture positivity KOH: potassium hydroxide

Figure 1 shows the total number of clinical samples according to KOH and culture positivity. For skin samples, there were 111 cases, with 28 testing positive for KOH (25.22%) and 32 testing positive for culture (28.82%). Nail samples included 73 cases, with 28 testing positive for KOH (38.35%) and 32 testing positive for culture (43.83%). In total, there were 184 clinical samples, with 56 testing positive for KOH (30.43%) and 63 testing positive for culture (34.23%). The chi-square test resulted in a p-value of 0.068 for skin samples. A p-value of less than 0.05 is considered significant (Figure 1).

Table 1 presents the descriptive statistics for the minimum inhibitory concentration (MIC) of five drugs, based on 184 samples. Amphotericin showed the lowest mean MIC (0.57 µg/mL, SD 0.87), followed by terbinafine (2.71 µg/mL, SD 6.00), griseofulvin (4.13 µg/mL, SD 6.20), itraconazole (3.38 µg/mL, SD 5.89), and fluconazole, which had the highest mean MIC (7.70 µg/mL, SD 14.39). Amphotericin and terbinafine achieved 100% inhibition, while itraconazole, fluconazole, and griseofulvin showed 80% inhibition. These statistics reflect the varying effectiveness of the drugs against the tested organisms (Table 1).

**Table 1:** Descriptive statistics for selected antifungal agents MIC 100% inhibition for amphotericin and terbinafine 80% inhibition for itraconazole, fluconazole, and griseofulvin MIC: minimum inhibitory concentration

Antifungal Agents	Participants	Mean (MICs)	Std. Deviation (MICs)
Amphotericin	184	0.56	0.86
Itraconazole	184	3.38	5.89
Fluconazole	184	7.69	14.38
Terbinafine	184	2.7	6
Griseofulvin	184	4.13	6.2

Table 2 highlights the association of potassium hydroxide (KOH) and culture outcomes with amphotericin's minimum inhibitory concentration (MIC), analyzed using chi-square tests. KOH positivity showed a significant correlation with higher MIC values (p=0.000), as most positive cases exhibited MIC levels of 1 µL or higher. Higher MIC values were observed with specific fungal isolates, particularly *T. rubrum* (MIC 2-4 µL), *T. mentagrophytes* (MIC 1-2 µL), and *T. tonsurans*. This demonstrates the influence of fungal presence and species on amphotericin's MIC levels (Table 2).

**Table 2:** Distribution and association of KOH and culture outcomes with amphotericin MIC levels among participants KOH: potassium hydroxide; MIC: minimum inhibitory concentration

Oucome Variables		Amphotericin MICs Levels							
		0.12 (µL)	0.5(µL)	1(µL)	2 (µL)	4 (µL)	Total	Chi-square	P-value
KOH	Negative	0	2	1	5	0	128	11.640	0.000
	Positive	1	2	15	36	1	56		
Culture	Contamination	0	0	0	0	0	90	83.97	0.000
	No fungal growth	0	0	0	0	0	31		
	T. mentagrophytes	0	0	9	14	0	23		
	T. rubrum	0	2	3	17	1	23		
	T. tonsurans	1	2	3	3	0	9		
	T. indotineae	0	0	1	7	0	8		

Table 3 examines the association of KOH positivity and culture outcomes with fluconazole's minimum inhibitory concentration (MIC), revealing significant correlations (p=0.000). KOH-positive samples exhibited higher MIC values, with concentrations of 8 µL or more common. Specific fungal isolates demonstrated varied MIC distributions: *T. mentagrophytes* and *T. indotineae* were associated with higher MIC levels (16-64 µL), while *T. rubrum* primarily showed MIC levels of 4-8 µL. This highlights that fungal presence and species significantly influence fluconazole's MIC (Table 3).

**Table 3:** Distribution and association of KOH and culture outcomes with fluconazole MIC levels among participants KOH: potassium hydroxide; MIC: minimum inhibitory concentration

Oucome Variables		Fluconazole MIC Levels							
		4(µL)	8(µL)	16(µL)	32(µL)	64(µL)	Total	Chi-square	P-value
KOH	Negative	0	3	1	2	2	128	4.424	0.000
	Positive	4	18	8	22	3	56		
Culture	Contamination	0	0	0	0	0	90	83.53	0.000
	No fungal growth	0	0	0	0	0	31		
	T. mentagrophytes	0	0	8	15	0	23		
	T. rubrum	4	19	0	0	0	23		
	T. tonsurans	0	2	1	2	4	9		
	T. indotinae	0	0	0	7	1	8		

Table 4 explores the association of KOH positivity and culture outcomes with griseofulvin's minimum inhibitory concentration (MIC), showing significant results (p=0.000). KOH-positive samples had higher MIC values, with the majority falling at 8 µL or 16 µL. Specific fungal isolates displayed distinct MIC patterns: *T. mentagrophytes* and *T. indotineae *were linked to an MIC of 16 µL, *T. rubrum* predominantly to 8 µL, and *T. tonsurans* exclusively to 8 µL. This underscores that fungal presence and species significantly affect griseofulvin's MIC (Table 4).

**Table 4:** Distribution and association of KOH and culture outcomes with griseofulvin MIC levels among participants KOH: potassium hydroxide; MIC: minimum inhibitory concentration

Oucome Variables		Griseofulvin MICs Level				
		8 (µL)	16(µL)	Total	Chi-square	P-value
KOH	Negative	6	2	128	2.439	0.000
	Positive	25	30	56		
Culture	Contamination	0	0	90	59.17	0.000
	No fungal growth	0	0	31		
	T. mentagrophytes	0	23	23		
	T. rubrum	22	1	23		
	T. tonsurans	9	0	9		
	T. indotineae	0	8	8		

Table 5 highlights the association of KOH positivity and culture outcomes with itraconazole's minimum inhibitory concentration (MIC), showing significant results (p=0.000). Higher MIC levels, with concentrations of 4-16 µL being more frequent. Specific fungal isolates demonstrated unique MIC patterns: *T. mentagrophytes* and *T. rubrum* showed higher MICs, with *T. rubrum* mostly at 16* µL, T. tonsurans* at 16 µL, and *T. indotineae* varying from 2-8 µL. This emphasizes the significant influence of fungal species on itraconazole's MIC (Table 5).

**Table 5:** Distribution and association of KOH and culture outcomes with itraconazole MIC levels among participants KOH: potassium hydroxide; MIC: minimum inhibitory concentration

Outcome Variables		Itraconazole MICs Level						
		0.5 (µL)	2(µL)	4(µL)	8(µL)	16(µL)	Chi-square	P-value
KOH	Negative	1	0	1	0	6	3.913	0.000
	Positive	7	1	11	13	23		
Culture	Contamination	0	0	0	0	0	66.90	0.000
	No fungal growth	0	0	0	0	0		
	T. mentagrophytes	8	0	8	7	0		
	T. rubrum	0	0	0	3	20		
	T. tonsurans	0	0	0	0	9		
	T. indotineae	0	1	4	3	0		

Table 6 examines the association of KOH positivity and culture outcomes with terbinafine's minimum inhibitory concentration (MIC), revealing significant results (p=0.000). KOH-positive samples showed higher MIC values, with most falling at 0.06 µL or 16 µL. Specific fungal isolates displayed distinct MIC patterns: *T. mentagrophytes* predominantly showed an MIC of 16 µL, *T. rubrum* clustered at 0.06 µL, *T. tonsurans* ranged from 0.03-0.12 µL, and *T. indotineae* was consistently at 16 µL. This underscores the significant impact of fungal presence and species on terbinafine's MIC (Table 6).

**Table 6:** Distribution and association of KOH and culture outcomes with griseofulvin MIC levels among participants KOH: potassium hydroxide; MIC: minimum inhibitory concentration

Outcome Variables		Terbinafine MICs Levels					
		0.03(µL)	0.06(µL)	0.12(µL)	16 (µL)	Chi-square	P-value
KOH	Negative	0	6	0	2	4.490	0.000
	Positive	4	20	1	29		
Culture	Contamination	0	0	0	0	68.176	0.000
	No fungal growth	0	0	0	0		
	T. mentagrophytes	0	0	0	23		
	T. rubrum	3	19	0	0		
	T. tonsurans	1	7	1	0		
	T. indotineae	0	0	0	8		

## Discussion

The findings of this study highlight critical insights into the antifungal susceptibility patterns of *Trichophyton* species, as determined by minimum inhibitory concentrations (MIC) using the broth microdilution assay. These results align with and expand upon existing literature, providing both supporting and contrasting perspectives on antifungal resistance trends and species-specific MIC variations.

Amphotericin's low mean MIC (0.57 µg/mL) and 100% inhibition align with earlier reports of its potent fungicidal activity against dermatophytes [9]. However, its higher MIC values in KOH-positive samples and specific fungal isolates like *T. rubrum* and *T. mentagrophytes* reflect potential variations in resistance patterns, possibly linked to geographic differences or prior antifungal exposure [5].

For Terbinafine, its excellent efficacy and 100% inhibition support previous findings of its strong action against dermatophytes. However, its elevated MIC values in *T. mentagrophytes* and *T. indotineae* are concerning, corroborating recent reports of terbinafine resistance in these species [6]. This underscores the importance of routine susceptibility testing to monitor resistance trends.

Fluconazole's high mean MIC (7.70 µg/mL) and only 80% inhibition are consistent with its known limited efficacy against dermatophytes (Gupta et al., 2020) [10]. The significantly higher MIC values observed in *T. mentagrophytes* and *T. indotineae* mirror recent studies documenting fluconazole resistance in these isolates (Nenoff et al., 2023) [11]. These findings suggest that fluconazole may no longer be a reliable option for treating infections caused by these resistant species.

Itraconazole showed moderate efficacy with an 80% inhibition rate and a mean MIC of 3.38 µg/mL. Its higher MIC levels in KOH-positive samples and isolates like *T. rubrum* and *T. tonsurans* align with findings of species-specific variability in susceptibility [9]. This highlights the potential role of itraconazole in managing infections caused by moderately resistant strains, although its effectiveness may vary by species.

Griseofulvin demonstrated moderate activity with a mean MIC of 4.13 µg/mL and 80% inhibition. Its higher MIC values in* T. mentagrophytes* and *T. indotineae* are consistent with reports of declining susceptibility in certain dermatophyte species [5]. This trend may be linked to evolving resistance mechanisms or pharmacokinetic challenges.

The absence of gender-based differences in MIC distributions across all drugs is consistent with previous studies indicating that antifungal susceptibility is not significantly influenced by gender [4].

The significant associations of KOH positivity and culture outcomes with higher MIC values across all drugs emphasize the influence of fungal load and species diversity on antifungal efficacy. Specifically, isolates like *T. mentagrophytes, T. indotineae*, and *T. rubrum* consistently exhibited higher MIC values, reflecting their potential role in driving resistance trends. This aligns with recent studies advocating for species-specific susceptibility testing to optimize treatment outcomes [6,11].

Study strengths

Clearly highlight the strengths of the study, such as the systematic use of standardized methods (CLSI) for antifungal susceptibility testing and its focus on *Trichophyton*, a significant dermatophyte genus.

Study limitations

The study evaluated only five antifungal agents, despite the existence of numerous other antifungal agents that also exhibit resistance. The samples were collected from patients who had received prior treatment, leading to a low culture yield. Out of 184 samples, only 63 culture isolates were available for antifungal susceptibility testing, which limits generalizability due to the sample size or geographical focus.

The resistance patterns of these additional agents should be investigated in future studies with alternative methodologies, including broader sample sizes and research designs, emphasizing the importance of species-specific testing and resistance monitoring to make the findings more relevant to healthcare practitioners. 

## Conclusions

In conclusion, this study sheds light on the intricate dynamics of antifungal resistance in dermatophytes, particularly concerning key antifungal agents such as fluconazole, griseofulvin, itraconazole, and terbinafine. MIC distributions play a critical role in understanding antifungal effectiveness in clinical microbiology. Results focused on unexpected resistance to typically effective drugs like terbinafine or fluconazole. As MIC-guided therapy improves outcomes by ensuring adequate drug exposure, the study results guide clinicians to choose the most appropriate antifungal for a given infection, minimizing toxicity and maximizing efficacy. These findings highlight the varying efficacy of antifungal agents against *Trichophyton* species and the critical role of species-specific and geographic resistance monitoring. Routine susceptibility testing and targeted antifungal stewardship are essential to mitigate resistance and improve therapeutic outcomes for dermatophytosis. 
